# Fuzzy-Based Approach Using IoT Devices for Smart Home to Assist Blind People for Navigation

**DOI:** 10.3390/s20133674

**Published:** 2020-06-30

**Authors:** Shahzadi Tayyaba, Muhammad Waseem Ashraf, Thamer Alquthami, Zubair Ahmad, Saher Manzoor

**Affiliations:** 1 Department of Computer Engineering, The University of Lahore, Lahore 54000, Pakistan; shahzadi.tayyaba@dce.uol.edu.pk; 2 Department of Physics (Electronics), Government College University, Lahore 54000, Pakistan; sahermanzoor1@gmail.com; 3 Electrical and Computer Engineering Department, King Abdulaziz University, Jeddah 21598, Saudi Arabia; tquthami@kau.edu.sa; 4 Center for Advanced Material (CAM), Qatar University, Doha P.O. Box 2713, Qatar

**Keywords:** IoT, smart home, fuzzy approach, ultrasonic sensors, bluetooth protocol, navigation

## Abstract

The demand of devices for safe mobility of blind people is increasing with advancement in wireless communication. Artificial intelligent devices with multiple input and output methods are used for reliable data estimation based on maximum probability. A model of a smart home for safe and robust mobility of blind people has been proposed. Fuzzy logic has been used for simulation. Outputs from the internet of things (IoT) devices comprising sensors and bluetooth are taken as input of the fuzzy controller. Rules have been developed based on the conditions and requirements of the blind person to generate decisions as output. These outputs are communicated through IoT devices to assist the blind person or user for safe movement. The proposed system provides the user with easy navigation and obstacle avoidance.

## 1. Introduction

The implementation of public health awareness has reduced the blindness cases due to diseases. But unfortunately, the rate of blindness in elderly people is high and increasing. These people need devices to assist them in navigation. This has increased the demand for assistive devices for navigation and orientation. The tools that are already available cannot provide all the information for safe mobility [1,2,3]. Vision substitution devices include three categories to improve blind people’s mobility. Each category slightly differs in features and actions from the other. The first category includes travel aids, which are the electronic devices that provide the user with information about the surroundings. This information helps the user develop a mental map for safe mobility. The second category includes orientation-based aids that provide the user with mobility instructions in unfamiliar places by defining and tracing the best route. Electronic devices in the third category are the position-based locators that use the global positioning system (GPS) technology to precisely locate the user. According to the needs of the user, the system or devices must have fast processing, large coverage with increased range detection of static and dynamic obstacles, and capacity to work equally well in day and night [4].

In the present era, wireless communication with wireless channels is a rapidly growing branch of technology. However, with such an emerging field, fast technological advances and developments are essential. Modern communication provides a wide range of services including data, voice, and multimedia, but the main issue in the communication field is to improve channel capacity. The channel capacity without interrupting the service quality can be enhanced by using multiple input and multiple output methods. It is one of the latest technologies and the estimation of data with this technology is done on the basis of maximum probability [5,6,7,8]. Bluetooth technology and ultrasonic sensors can be used in modern technology with the ability to communicate along with direct access to the fixed infrastructure. The sensors can sense any obstacles or deviations that may be static or dynamic. With the help of a navigation system, the target can be located easily [9]. Utilization of IoT-based devices has various advantages to be used in emerging technologies. Efficacy, compatibility, and consistency on a global scale result in the success of IoT. A combination of various technologies along with software can result in a hybrid system with applications in various fields of interest in communication [10,11].

In behavior-based navigation, each behavior develops sensory information and transforms it into a response. The problem with such a system is that several commands may be produced simultaneously with multiple behaviors, which may cause the system to fail, while fuzzy control systems are based on IF-THEN rules [12]. The implementation of fuzzy-based methods in the case of adaptive techniques provides the fast convergence and reduced complexity in conditions that are nonlinear and vary with time. A fuzzy approach is highly suitable for the incorporation of human expert knowledge to balance already-available numerical data. Amongst several artificial intelligence techniques, fuzzy logic is considered a useful tool in the navigation control system for its linguistic terms and reliable decision-making capability without precise information of the surroundings. It utilizes human reasoning and decision making for reliable navigation in a dynamic environment with unknown obstacles [12,13]. Two factors are crucial for the safe mobility of blind people, which are path tracking and obstacle avoidance. Fuzzy logic can be used for the development of such systems. 

A variety of studies have been carried out on fuzzy logic to develop path tracking for vehicles, adjust speed and direction of vehicles according to present and future path information, designing and implementation of path tracking in indoor environment, autonomous path following, obstacle avoidance by taking distance, and change in distance from the obstacle as input parameters. The output parameter in these cases is the speed of the obstacle. A comparison of obstacle avoidance by mobile robots with Sugeno and Mamdani fuzzy logic controller have also been reported. Additionally, for obstacle detection, ultrasonic as well as infrared (IR) sensors have been widely used in literature. IR sensors are based on sound sensor and cannot operate under dark condition, whereas ultrasonic sensors have linear output characteristics and can detect all types of obstacles [14,15,16,17,18,19,20,21,22].

In this research work, the crucial parameters for the safe mobility of blind people have been considered using an IoT-based system for a smart home model. Using fuzzy logic controller, a reliable device for obstacle avoidance, whether static or dynamic, along with target tracking has been proposed. Fuzzy-based simulation has been verified with Mamdani model calculations for result comparisons and optimizations. 

## 2. Description of System for Navigation to Assist Blind People

**Figure 1:**
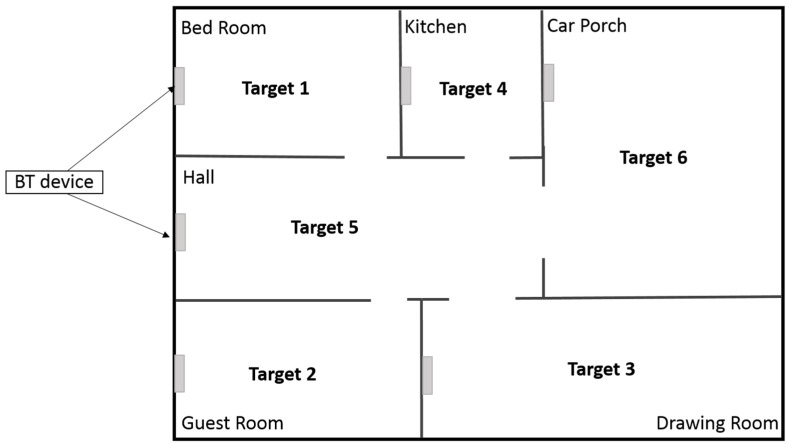
Model of a smart home for a blind person.

A model for smart home is created using IoT based system, which consists of sensors and antenna for receiving and transmitting signals. Bluetooth devices are installed in every room and the blind person is wearing a watch. The watch with the help of multiple ultrasonic sensors detects static or dynamic obstacles and gives audio signals to its user for navigation. When the user enters a certain room, a bluetooth device connects with the watch and informs the user about its location. The model of a smart home is shown in Figure 1. 

Bluetooth devices work in a certain manner from the transmitting and receiving antennas. A small chip is installed in the devices which receive or transmit signals. If ht is the height of the transmitting antenna and hr is the height of the receiving antenna with lambda (λ) as the wavelength, then the distance between the antennas is given by Equation (1) [23].

(1) $$d=\frac{4\left(ht.hr\right)}{\lambda }\;$$

After the calculation of the distance between the receiver and transmitter, the received signal strength indication can be done by Equation (2).

(2) RSSI=[log(d−20)]−a

where a represents the offset based on the maximum strength of the received signal and RSSI is the received signal strength indicator. The received signal at the receiver antenna is given by Equation (3).

(3) $$Rb\left(i\right)=\overset{X}{\underset{x=1}{\sum }}hx.y\;dx\left(i\right)+vy\left(i\right)\;$$

where Rb is the received signal at receiver, the first term of the right side of the equation indicates the coefficient relating to the receiver and transmitter antenna and the second term represents the noise factor. The normalized channel gain is given by Equation (4) [24].

(4) $$\overset{X}{\underset{x=1}{\sum }}\left|hx,y\right|{\;}^{2}=1$$

**Figure 2:**
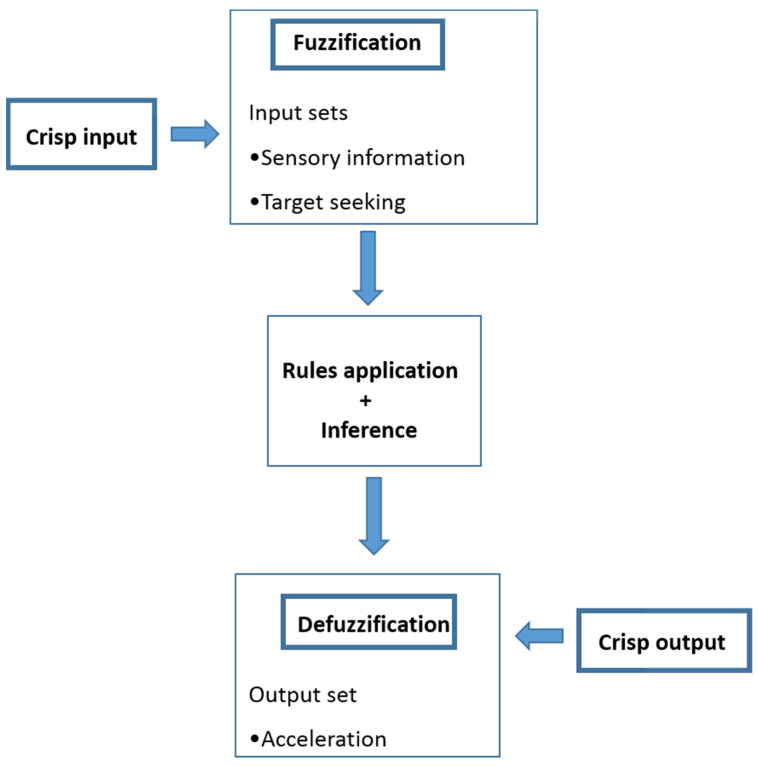
Schematic for the Fuzzy Logic Controller.

Fuzzy logic controller (FLC) is used to operate and navigate the whole system. The outputs of the sensors are provided as inputs to the controller and it makes decision making on the basis of rules according to the information contained by the output of the sensors. The command from the FLC is sent to audio devices for generation of audio signals to navigate the user in a smart home along with the warning for any obstacles in the surroundings. The schematic for FLC is shown in Figure 2. First of all, fuzzification of the inputs is done followed by applying the rules. After defining the rules, the output is generated by the defuzzification of inputs using a fuzzy interface. 

**Figure 3:**
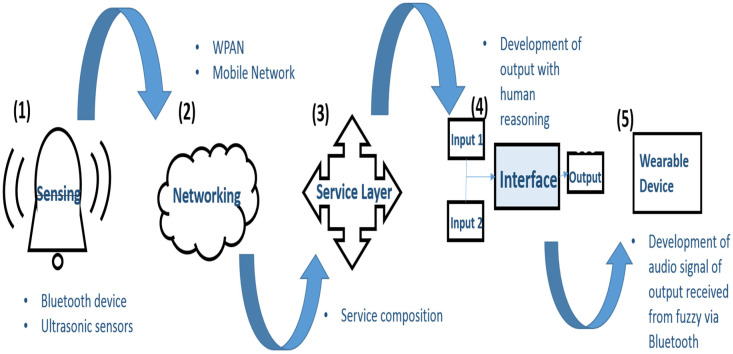
System flow diagram for final output generation.

This system works on the basis of three inputs and two outputs. The output of ultrasonic sensors and target direction has been taken as input parameters. The rules for the input and output are generated based on options’ availability (IF circumstances THEN action). First of all, a top-down analysis of the scenario is done for task development, then, bottom-up commands are generated to assess the validity of the task before its actual execution. For instance, if the input is no obstacle towards the right and target towards the right, then the output generated will be “turn right”. However, if the input is obstacles towards the right and the target is also towards the right, then the output generated will be “keep moving forward”. The safety of the user is the priority of the system. The complete working strategy of our system is a hybrid setup consisting of IoT devices along with a fuzzy interface. The working strategy of the device is shown in Figure 3. The outputs from the sensing and networking devices are supplied to the interface as input, and finally, output is generated using human reasoning. The output from the fuzzy logic is then transferred to the wearable device via bluetooth and is converted to an audio signal.

## 3. Simulation and Results

**Figure 4:**
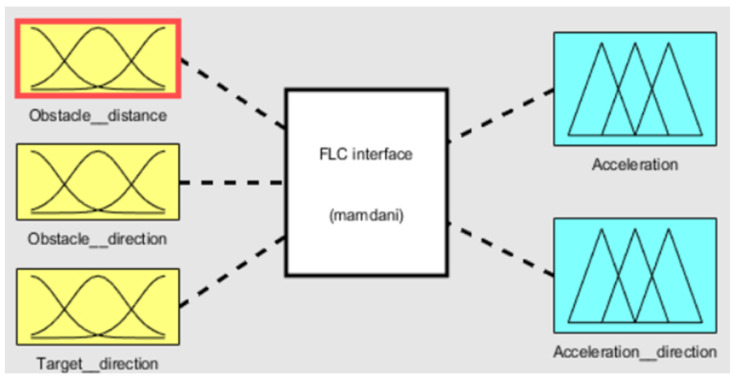
Fuzzy interface with three inputs and two outputs.

Three parameters are selected as input variables with two corresponding outputs. Obstacle distance, obstacle direction, and target direction are the three inputs in the FLC interface with acceleration and direction of acceleration as output. Figure 4 shows the fuzzy interface using Mamdani’s model [25] with input and output parameters.

**Figure 5:**
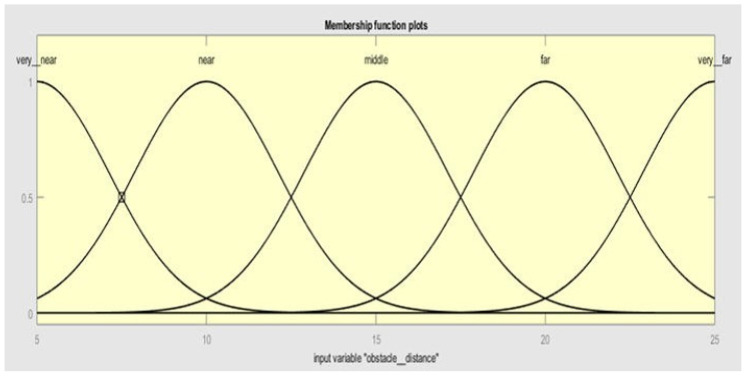
Membership functions for input 1 (obstacle distance).

Five membership functions are selected for obstacle distance. As shown in Figure 5, the membership functions are *very__near, near, middle, far, and very__far*.

First of all, the location of the user is determined and then the distance from obstacles and targets will be calculated. The values for the inputs are given by Equation (5).

(5) $$r=\sqrt{{\left(x_{2}-x_{1}\right)}^{2}+{\left(y_{2}-y_{1}\right)}^{2}}$$

where r represents the distance between two points, $x_{2}-x_{1}$ is the change in the x coordinate, and $y_{2}-y_{1}$ is the change in the y coordinate.

**Figure 6:**
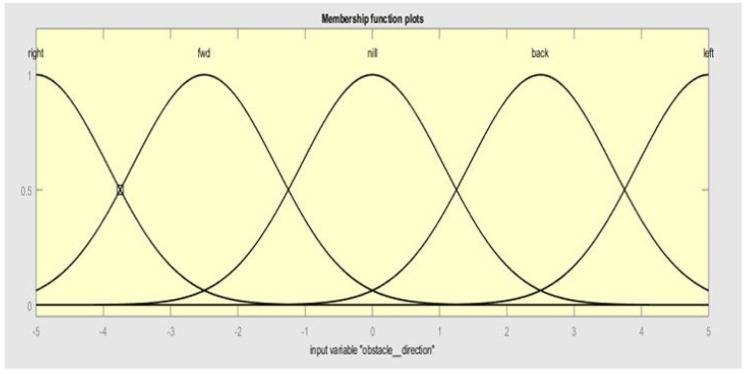
Membership functions of input 2 (obstacle direction).

OR, AND, and NOT connectives are normally used for fuzzy compound preposition. OR represents union, AND represents intersection, and NOT is complement. If variables for obstacle distance, obstacle direction, target direction, acceleration, and acceleration direction are represented by OD, ODir, TD, A, and AD, then the preposition for fuzzy is represented by *T: OD × ODir × TD = A, AD.* Similarly five membership functions of input 2 (obstacle direction) are right, fwd, nill, back, and left, as shown in Figure 6.

**Figure 7:**
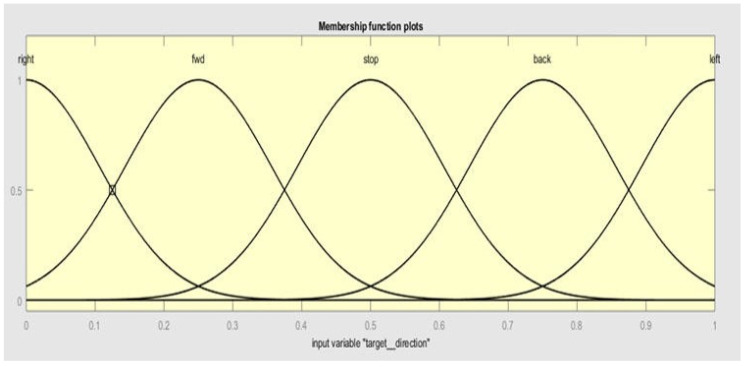
Membership functions of input 3 (target direction).

Finally, for input 3 (target direction), the membership functions are taken as right, fwd, stop, back, and left, as shown in Figure 7. 

**Figure 8:**
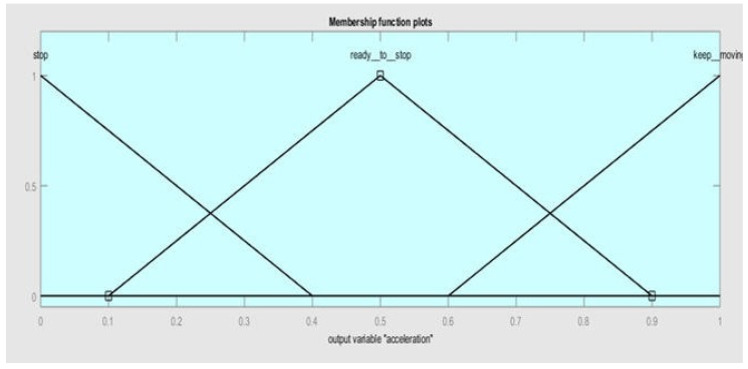
Membership functions of output 1 (acceleration).

The membership functions for output 1 (acceleration) are *stop, ready__to__stop, and keep__moving*, as shown in Figure 8. 

**Figure 9:**
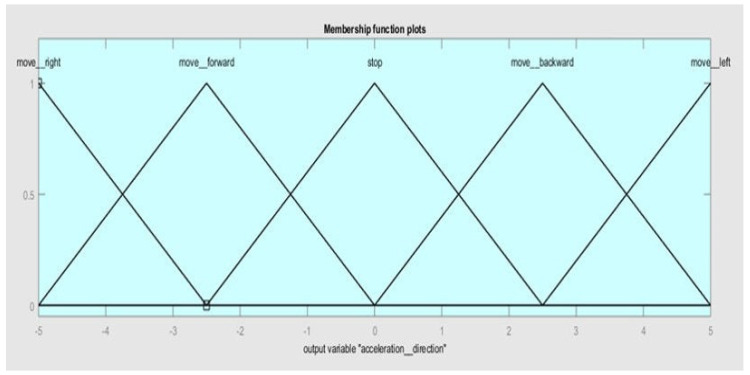
Membership functions of output 2 (acceleration direction).

Finally, the membership functions for output 2 (acceleration direction) are *move__right, move__forward, stop, move__backward, and move__left*, as given in Figure 9.

**Figure 10:**
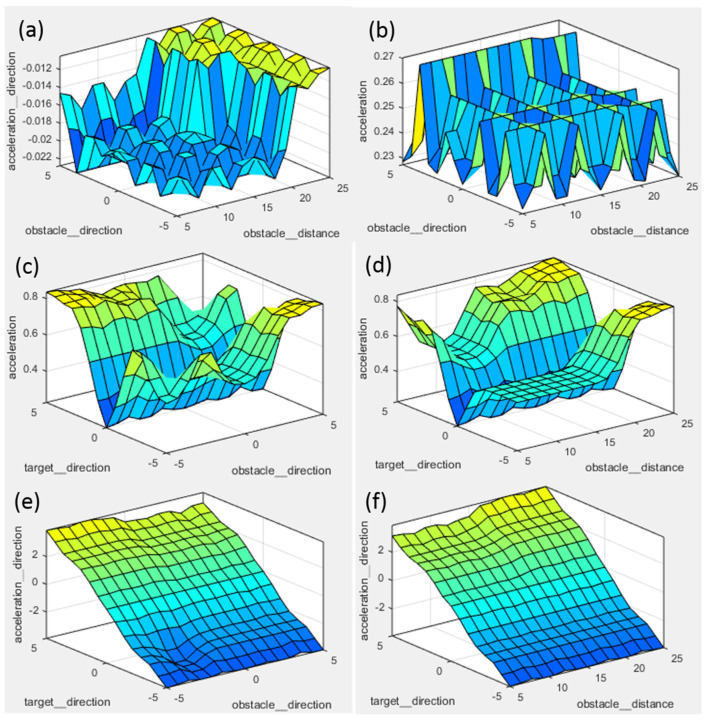
3D graphs for inputs and outputs. (**a**) obstacle direction, obstacle distance versus acceleration direction; (**b**) obstacle direction, obstacle distance versus acceleration; (**c**) target direction, obstacle direction versus acceleration; (**d**) target direction, obstacle distance versus acceleration; (**e**) target direction, obstacle direction versus acceleration direction; and (**f**) target direction, obstacle distance versus acceleration direction.

As all three inputs have five membership functions, therefore, the rules for the output generated will be calculated as 5 × 5 × 5 = 125 using the Mamdani model formula. Therefore, 125 rules are generated using an IF and THEN formulation. For example, IF obstacle distance is very far and obstacle direction is left and the target direction is right, THEN acceleration is keep moving and acceleration direction is right. For decision making, the priority is given to the safety of the user. The three-dimensional graphs of the two inputs with different combinations are plotted against the two outputs, as shown in Figure 10.

**Figure 11:**
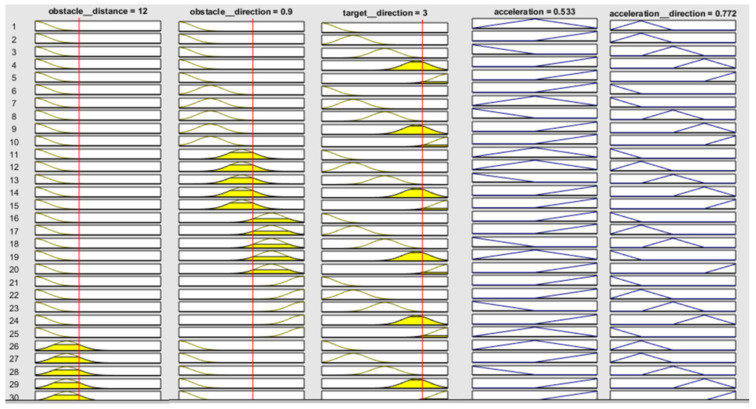
Rule viewer plot.

Mamdani’s model has been used for error estimation and comparison of simulation results. Rule viewer graph is shown in Figure 11. From this rule viewer, fuzzy values are selected and further calculations are done.

Fuzzy values for all the three inputs are selected. For obstacle distance, the value selected is 12, for obstacle direction, the value is 0.9, and for target selection, the value is 3. After that, the values of membership functions $y_{1},\;y_{2},\;y_{3},\;y_{4},y_{5}\;and\;y_{6}$ are calculated with the help of Mamdani’s model by the Equations (6) and (7).

$y_{1}\;and\;y_{2}$ are calculated as: max value of input 1—crisp value/max value.

(6) $$y_{1}=\frac{25-12}{25}=0.52$$

(7) $$y_{2}=1-y_{1}=0.48$$

Similarly, the values for $y_{3},\;y_{4},\;y_{5},\;and\;y_{6}$ are calculated as 0.82, 0.18, 0.4, and 0.6, using maximum value and crisp value for input 2 and input 3, respectively. For calculation of outputs acceleration and acceleration direction, Table 1 and Table 2 are presented with some suitable rules.

The singleton values are calculated by dividing the output values corresponding to selected input values by 100.

The Mamdani model is implemented here and represented in Equation (8).

(8) $$Mamdani\;Model=\left[\sum \left(M_{i}\times S_{i}\right)\right]/\sum M_{i}\;$$

By using the above formula, calculations have been performed for the minimum singleton values and we calculated Σ Mi=2.52,  Σ (Mi×Si1)=0.015, using Mamdani’s Model=[Σ (Mi×Si1) / ΣMi ]×100=0.6. The simulated MATLAB value is 0.53. The same has been done for output 2 (acceleration direction). It has been found that the minimum and singleton values are Σ Mi=2.52, Σ (Mi×Si2)=0.02, respectively. The Mamdani expression becomes [Σ (Mi×Si2) / ΣMi ]×100=0.79 and the simulated MATLAB value is 0.77.

From the above results, it is clear that the simulated values and calculated values based on the Mamdani model are very close. Hence, IoT-based systems are useful with the fuzzy-based approach to assist blind people for safe movement in a smart home [26,27]. The presented work would provide handy information for the development of a real-time efficient and reliable system for people who cannot survive independently in normal circumstances with ease. In a future work, the implementation of this model will be performed by considering additional vibrational signals, electronic circuits, and IoT device integration. With some extension, the system can be used to provide support to the deaf person, for ease in navigation and communication.

## 4. Conclusions

Here, a model for smart home using the IoT system is proposed. The IoT system comprising sensors and antennas generates warning signals about the obstacles in the way of users and also navigates the user to move around the house safely. The outputs from the IoT system are used as inputs in the FLC. Thus, in case of multiple behavior inputs, the decision is made with human reasoning and on the basis of likelihood. Fuzzy-based simulation has been carried out. Calculated results are compared with simulated values. It shows accurate processing of data, reliability, and mobility of the blind user indoor.

## Figures and Tables

**Table 1:** Calculations for acceleration.

Rule No.	Obstacle Distance	Obstacle Direction	Target Direction	Acceleration (a)	Member Ship Function Values (MFs Values)	Min. Value of MFs (Mi)	Singleton Value for Acceleration (Si1)	Mi × Si1
R1	Very near	Fwd	Back	Keep moving	y1^y3^y5	0.4	0.01	0.004
R2	Very near	Fwd	Stop	Stop	y1^y3^y6	0.52	0	0
R3	Near	Back	Right	Keep moving	y1^y4^y5	0.18	0.01	0.0018
R4	Near	Back	Fwd	Keep moving	y1^y4^y6	0.18	0.01	0.0018
R5	Middle	Right	Back	Keep moving	y2^y3^y5	0.4	0.01	0.004
R6	Middle	Right	Stop	Stop	y2^y3^y6	0.48	0	0
R7	Far	Back	Left	Keep moving	y2^y4^y5	0.18	0.01	0.0018
R8	Far	Left	Back	Keep moving	y2^y4^y6	0.18	0.01	0.0018

**Table 2:** Calculations for acceleration direction.

Rule No.	Obstacle Distance	Obstacle Direction	Target Direction	Acceleration Direction	Member Ship Function Values (MFs Values)	Min. Value Of MFs (Mi)	Singleton Value for Acceleration Direction (Si2)	Mi × Si2
R1	Very near	Fwd	Back	Move Back	y1^y3^y5	0.4	0.025	0.01
R2	Very near	Fwd	Stop	Stop	y1^y3^y6	0.52	0	0
R3	Near	Back	Right	Move right	y1^y4^y5	0.18	−0.05	−0.009
R4	Near	Back	Fwd	Move fwd	y1^y4^y6	0.18	−0.025	−0.0045
R5	Middle	Right	Back	Move back	y2^y3^y5	0.4	0.025	0.01
R6	Middle	Right	Stop	Stop	y2^y3^y6	0.48	0	0
R7	Far	Back	Left	Move left	y2^y4^y5	0.18	0.05	0.009
R8	Far	Left	Back	Move back	y2^y4^y6	0.18	0.025	0.0045
